# Bed bug deterrence

**DOI:** 10.1186/1741-7007-8-117

**Published:** 2010-09-09

**Authors:** Kenneth F Haynes, Mark H Goodman, Michael F Potter

**Affiliations:** 1 Department of Entomology, S-225 Agricultural Science Center, University of Kentucky, Lexington KY 40546, USA

## Abstract

A recent study in *BMC Biology *has determined that the immature stage of the bed bug (the nymph) signals its reproductive status to adult males using pheromones and thus avoids the trauma associated with copulation in this species. The success of this nymphal strategy of deterrence is instructive. Against the background of increasing problems with bed bugs, this research raises the question whether pheromones might be used to control them.

See research article http://www.biomedcentral.com/1741-7007/8/121

## Bed bug resurgence

A global resurgence of bed bugs, a once common household pest that had nearly disappeared for 50 years, has renewed scientific interest in these fascinating insects. Pest control companies from every part of the world are reporting many more encounters now than 10 years ago [1]. Undoubtedly, many factors have led to this outbreak, but evolved resistance to some of the most commonly used insecticides is a contributor [2]. Unlike many other blood-feeding arthropods, such as mosquitoes, tstetse flies, ticks, and sand flies, bed bugs are not known to effectively vector any human pathogen. However, bed bugs inhabit our beds, and return repeatedly at night [3] for blood meals required to complete each stage of development (five immature stages) and each cycle of egg production. Recent research by Harraca and colleagues presented in *BMC Biology *provides new insights into the reproduction of bed bugs that may offer an untapped opportunity for pest control [4].

## Anti-aphrodisiac pheromones defend vulnerable nymphs

Harraca and colleagues have demonstrated that bed bug nymphs (*Cimex lectularius*) produce a chemical signal that interrupts the attempts of adult males to mate with them [4]. Because adult males, females, nymphs and eggs are found in aggregations around where the host sleeps (in the case of humans, our beds), encounters between males and nymphs are common. Copulation between an adult and a nymph is reproductively ineffective, but can be very costly to the nymph and the male; rupture of the cuticle for the nymph, and loss of sperm and other components of the ejaculate for males. As a result the reproductive fitness of the male and survival of the nymphs are parallel interests. These are exactly the circumstances that should favour the evolution of communication, because both signaller and receiver benefit from the information transfer. Two complementary manipulative experiments conducted by Harraca *et al*. [4] provide convincing evidence of effective communication between nymphs and males. When the glandular source of the scent that is unique to nymphs is blocked, males will copulate with them. When a nymph-specific compound or nymph-specific ratio of compounds were puffed on male-female pairs, mating was disrupted. Furthermore, males have sensory neurons that respond to the nymph odours. Thus, the chemical signal translates into the simple received message that the source nymph is not a reproductive female. The nature of copulation, known as traumatic or hypodermic insemination, may help to explain the evolution of communication between nymphs and males.

## Traumatic insemination

**Figure 1:**
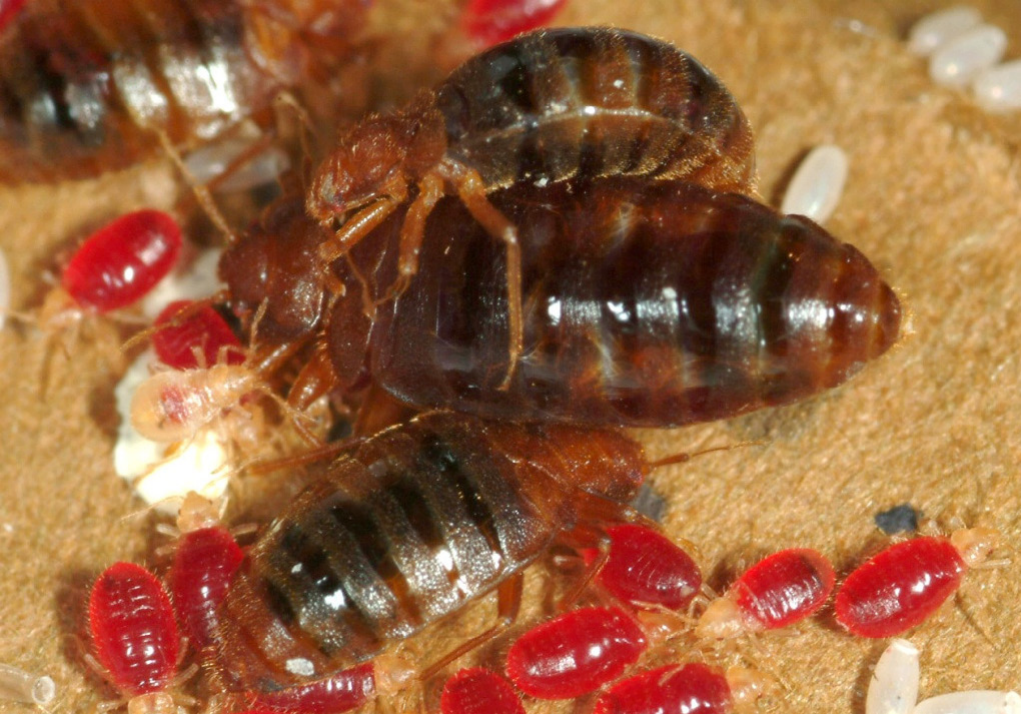
**Mating pair (male top) in aggregation of recently fed bed bugs**.

**Figure 2:**
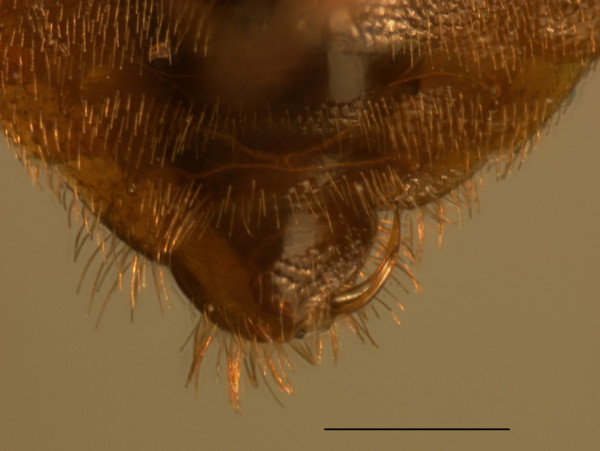
**The sickle-shaped paramere at the end of the males abdomen penetrates the cuticle of the female and introduces sperm and accessory gland fluids into her body cavity**. Ventral view. Horizontal bar is 5 mm.

Reproductive modes of insects are remarkably diverse, as one might expect from a class of animals with millions of species, with some found in every terrestrial habitat, and most aquatic environments as well. Within that diversity, traumatic insemination stands out as an evolutionary enigma. It is a characteristic of the family Cimicidae, a family to which *C. lectularius *(the bed bug or the human bed bug), *C. hemipterus *(the tropical bed bug, another human parasite), and over 70 species that are ectoparasites of birds and bats belong [5]. During copulation the male curls his abdomen under the female (Figure 1), punctures her cuticle with a rigid, sickle-shaped paramere (Figure 2) and introduces sperm and accessory gland fluids into her body cavity [6-10]. Sperm must migrate through the haemocoel to the female's reproductive system. The extragenital adaptations seen in females greatly reduce the costs that would be associated with punctures of the cuticle [6]. Even with these modifications, mating is costly, occurring with an excessive frequency that is roughly 20 times that necessary for fertilization of eggs [9]. Each copulation increases risk of infection or physical damage and because nymphs are immature and lack the external and internal adaptations they are at even greater risk.

## Adaptations to traumatic insemination in mature bedbugs

**Figure 3:**
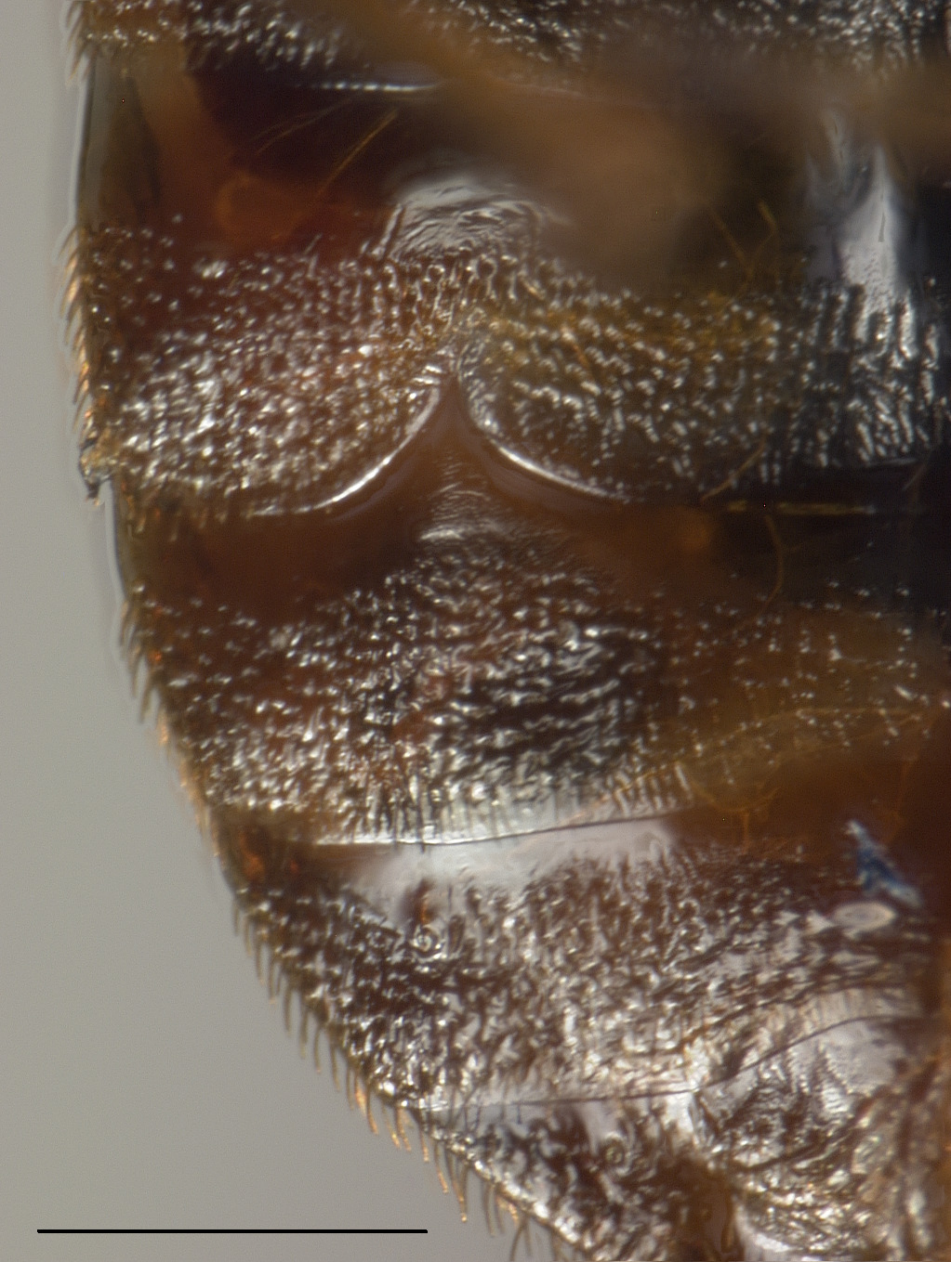
**The ectospermalege (inverted V-shape) is an extragenital cuticular structure only on the right side of female**. By channelling the male paramere to this part of the abdomen it limits the damage done. Ventral view. Horizontal bar is 5 mm. Internally the female has a mesospermalege that receives sperm and has a presumed function in the immune responses of the female.

In sexually reproducing organisms, the reproductive interests of males and females are not necessarily congruous. Often males can increase their fitness by mating with many females, but female success may be optimized by mating with high quality sires. Traumatic insemination represents an extreme in the conflict of interests of males and females [11]. *Primicimex cavernis *resembles a common ancestor of all Cimicidae with respect to copulation; it has traumatic insemination, but not the female counter-adaptations seen in most other species [5]. In contrast, *C. lectularius *has an asymmetrical ectospermalege (right-side only; Figure 3) that channels the sickle-shaped male paramere to puncture the cuticle so that semen is introduced into the mesospermalege, which is an internal structure with presumed immunological function. The internal adaptations are even more extensive in other species of Cimicidae. In addition to morphological and immunological adaptations, females reduce extra copulations by dispersing from aggregations [11]. For an insect that benefits from proximity to host and microhabitat humidity control, which is enhanced by aggregation [12], dispersal carries its own risks. The evolution of insemination that bypasses the genital system and the subsequent evolution of an extragenital system in the female is an example of the coevolution mediated by sexual conflict [6-11]. Males also have adaptations to avoid injury from homosexual copulation, such as release of a blend of chemicals from the metathoracic gland [9], which in a different context has a putative alarm function [13].

## An example to follow?

It remains to be seen if the newer thrusts of basic science research, such as that reported by Harraca and colleagues in *BMC Biology*, will contribute new approaches to pest control, but certainly the reliance of bed bugs on chemical signalling [13-15] is one avenue that needs to be explored further. Interfering with mating behaviour is an attractive idea, which has shown success with many agricultural pest species, but the longevity of bed bugs (months to a year or more) and our lack of tolerance of their repeated biting, makes the concept much more challenging. Whether interfering with sexual reproduction with 'anti-aphrodiasiac' pheromones is of practical significance or not, ongoing research on bed bug biology is likely to have an impact on the way that we manage this tough pest.

## References

[B1] Potter MF Rosenberg B Henriksen M Bugs without borders: defining the global bed bug resurgence Pest World 2010 September/October 8 20

[B2] Romero A Potter MF Potter DA Haynes KF Insecticide resistance in the bed bug: A factor in the pest's sudden resurgence? J Med Entomol 2007 44 175 178 10.1603/0022-2585(2007)44[175:IRITBB]2.0.CO;2 17427684

[B3] Romero A Potter MF Haynes KF Circadian rhythm of spontaneous locomotor activity in the bed bug, *Cimex lectularius *L J Insect Physiol 2010 56 1516 1522 10.1016/j.jinsphys.2010.04.025 20452356

[B4] Harraca V Ryne C Ignell R Nymphs of the common bed bug (*Cimex lectularius*) produce anti-aphrodisiac defence against conspecific males BMC Biology 2010 8 121 10.1186/1741-7007-8-121 PMC2944131 20828381

[B5] Usinger RL Monograph of Cimicidae (Hemiptera, Heteroptera) 1966 College Park, MD: Entomological Society of America

[B6] Morrow EH Arnqvist G Costly traumatic insemination and a female counter-adaptation in bed bugs Proc R Soc B 2003 270 2377 2381 10.1098/rspb.2003.2514 14667354 PMC1691516

[B7] Reinhardt K Naylor R Siva-Jothy MT Reducing a cost of traumatic insemination: female bedbugs evolve a unique organ Proc R Soc B 2003 270 2371 2375 10.1098/rspb.2003.2515 14667353 PMC1691512

[B8] Reinhardt K Siva-Jothy MT Biology of the bed bugs (Cimicidae) Annu Rev Entomol 2007 52 351 374 10.1146/annurev.ento.52.040306.133913 16968204

[B9] Siva-Jothy MT Trauma, disease and collateral damage: conflict in cimicids Phil Trans R Soc Lond B Biol Sci 2006 361 269 275 10.1098/rstb.2005.1789 PMC1569606 16612886

[B10] Stutt AD Siva-Jothy MT Traumatic insemination and sexual conflict in the bed bug *Cimex lectularius* Proc Natl Acad Sci USA 2001 98 5683 5687 10.1073/pnas.101440698 11331783 PMC33273

[B11] Pfiester M Koehler PG Pereira RM Sexual conflict to the extreme: traumatic insemination in bed bugs Amer Entomol 2009 55 244 249

[B12] Benoit JB Del Grosso NA Yoder JA Denlinger DL Resistance to dehydration between bouts of blood feeding in the bed bug, *Cimex lectularius*, is enhanced by water conservation, aggregation, and quiescence Am J Trop Med Hyg 2007 76 987 993 17488928

[B13] Levinson HZ Levinson AR Maschwitz U Action and composition of alarm pheromone of bedbug *Cimex lectularius *L Naturwissenschaften 1974 61 684 685 10.1007/BF00606522 4449574

[B14] Ryne C Homosexual interactions in bed bugs: alarm pheromones as male recognition signals Anim Behav 2009 78 1471 1475 10.1016/j.anbehav.2009.09.033

[B15] Siljander E Gries R Khaskin G Gries G Identification of the airborne aggregation pheromone of the common bed bug, *Cimex lectularius* J Chem Ecol 2008 34 708 718 10.1007/s10886-008-9446-y 18470566

